# Global re-emergence of human monkeypox: Population on high alert

**DOI:** 10.3126/nje.v12i2.45974

**Published:** 2022-06-30

**Authors:** Indrajit Banerjee, Jared Robinson, Brijesh Sathian

**Affiliations:** 1, 2 Sir Seewoosagur Ramgoolam Medical College, Belle Rive, Mauritius; 3 Geriatric and long term care Department, Rumailah Hospital, Hamad Medical Corporation, Doha, Qatar

## Background

Monkey pox is a rare zoonotic viral disease and is a member of the Orthopoxvirus genus in the family Poxviridae. Monkeypox is closely related to the only worldwide eradicated disease Smallpox (variola virus). Monkeypox is however a milder and less severe disease as compared to its cousin Small pox which was officially declared eradicated on the 8th of May 1980 at the thirty third World Health Assembly. Monkey pox has since found itself thrusted into the limelight of the international media and health authorities as numerous outbreaks have been recorded in non-endemic countries. If the aforementioned outbreaks of the monkey pox disease are not swiftly and actively controlled, the international community may find itself battling not only the current global pandemic (COVID-19) but a possible epidemic within a pandemic [1,2].

### Monkey pox a brief history

The Monkey pox virus is a member of the Orthopoxvirus genus in the family “Poxviridae”. The Orthopoxvirus genus includes Monkey pox, Smallpox (variola virus), the vaccinia virus and the Cowpox virus. The first human case of Monkey pox was recorded in the 1970’s in the (DRC) Democratic Republic of the Congo. The virus was however first discovered earlier in primates in 1958, where a disease similar to pox in its characteristics and presentation broke out in a troop of monkeys which were kept for research activities and purposes. The outbreak in the primates prompted the nomenclature of “Monkey pox” to be coined [3].

Subsequent to its discovery, Monkey Pox has since become an endemic disease within the African continent, but namely confined to countries such as Liberia, Benin and Nigeria [4].

### Monkeypox virus

The Monkeypox virus belongs to the DNA group of viruses and can be divided into two monophyletic biological groups, namely the West African group and the Central African or (Congo basin) group. These two distinct monophyletic groups of the Monkeypox virus have varying case fatality (CFR) and transmission rates. The West African group demonstrates a (CFR) case fatality rate of less than 1%, and exhibits no human to human transmission. This is however starkly juxtaposed by the Central African (Congo basin) group which exhibits a (CFR) case fatality rate of up to near 11% and causes a disease of greater severity which is transmissible from human to human [5].

### Monkey Pox outbreak 2022

Numerous cases of Monkey Pox have been reported to the WHO since the 1st of January 2022. As of the 15th of June 2022, a total of two thousand one hundred and three (2103) confirmed cases and 1 death have been registered by the WHO. A single confirmed case of Monkey Pox in a nonendemic country officially constitutes as an outbreak. Monkey Pox cases have been reported to the WHO across 5 of its regions (The Western Pacific, Eastern Mediterranean, Europe, Africa and the Americas) and in forty-two of its member states. This outbreak of the Monkey Pox virus is being recorded and reported with sustained transmission between men who have engaged in sexual intercourse with men. As of 15^th^ June 2022, United Kingdom has the highest number of 524 registered cases followed by Spain with 313 cases, Germany 263 cases, Portugal 241 cases and Canada with 159 cases respectively. The WHO have assessed the risk of this current outbreak and have classed this Monkey Pox outbreak as a moderate health risk, mainly due to the low risk of death and mortality associated with the infection [5,6].

### Transmission of Monkey Pox

Transmission of the Monkey Pox virus occurs through direct contact. Close contact such as that of skin to skin, mouth to mouth and face to face with the exposure to open lesions or lesions of the buccal mucosa aid the transmission of the disease. The virus is also transmitted through contaminated objects and materials ranging from pillows to bedding and the like. The occurrence and spread of this most recent outbreak of the virus has been mainly attributed to the sexual route of transmission. The sexual transmission predominantly occurring in men whom have had sexual relations with other men. The majority of these cases reporting to their local primary healthcare centers with the relative signs and symptoms [7].

### Monkey Pox signs and symptoms

Cases infected with Monkey Pox present with a high fever (pyrexia), vesicular and pustular rashes on the palms and soles and lymphadenopathy (lymphadenopathy being the main differentiating fever between Small Pox and Monkey Pox). The presentation of Monkey Pox is flu-like in nature as with most infections of the viral sort [8].

### Treatment

Currently no licensed treatments are available for individuals infected with Monkey Pox, but brincidofovir and tecovirimat have shown promising use and efficacy against the virus in animals. The treatment is predominantly of the symptomatic type including paracetamol to combat the viral fever. Various medicaments can be used to prevent and control the Monkey Pox outbreak including (VIG) Vaccinia immunoglobulin, cidofovir and the Small Pox vaccine [8].

### Vaccination against Monkey Pox

The use of the variola vaccine (which was used to eradicate Smallpox) offers use against the Monkeypox virus. It is however used on an off-label basis to control minor outbreaks. The issue in the global supply of the vaccines is however availability, as the Small pox vaccination program was terminated after the eradication of the virus in the 1980’s. The Small pox vaccines currently in existence are grouped into first, second and third generation vaccines. The first generation being unsuitable for use against Monkey Pox as they are of the original vaccines used in the 1980’s to eradicate Small Pox. The newer generations of Small Pox vaccines however are indicated to be used for the prevention of Monkey Pox, MVA-BN being one of the preferred vaccines to be used against Monkey Pox. The greatest global tool available in the worlds’ arsenal to combat the viral infection will be through rigorous surveillance and rapid case detection to further hinder and control the spread of the disease [9,10].

## Conclusion

The current international outbreak of Monkey Pox, is by no means the next SARS-CoV-2 pandemic, however through stronger global surveillance and international collaboration the current up flaring of cases can be swiftly controlled. It is vital that such outbreaks of this virus induce health agencies to begin further research into the Poxviridae family and formulate newer and more effective specific treatments to adequately treat and prevent such events in future.

## References

[1] Otu A Ebenso B Walley J Barceló JM Ochu CL. Global human monkeypox outbreak: atypical presentation demanding urgent public health action. The Lancet Microbe. 2022 Jun 7. https://doi.org/10.1016/S2666-5247(22)00153-7 10.1016/S2666-5247(22)00153-7 PMiD: 35688169 PMC9550615

[2] Ligon BL. Monkeypox: a review of the history and emergence in the Western hemisphere. Semin Pediatr Infect Dis. 2004 Oct;15(4):280-7. https://doi.org/10.1053/j.spid.2004.09.001 10.1053/j.spid.2004.09.001 PMiD: PMCid: 15494953 PMC7129998

[3] About Monkeypox. [online] 2022 [cited 2022 June 20]. Available from URL: https://www.cdc.gov/poxvirus/monkeypox/about.html

[4] Kozlov M. Monkeypox goes global: why scientists are on alert. Nature. 2022;606(7912):15-6. https://doi.org/10.1038/d41586-022-01421-8 10.1038/d41586-022-01421-8 PMiD: 35595996

[5] Global outbreaks of monkeypox. [online] 2022 [cited 2022 June 20]. Available from URL: https://gvn.org/global-outbreaks-of-monkeypox/

[6] Multi-country monkeypox outbreak: situation update. World Health Organization (17 June 2022). [online] 2022 [cited 2022 June 20]. Available from URL: https://www.who.int/emergencies/disease-outbreak-news/item/2022-DON393

[7] Nolen LD Osadebe L Katomba J. Extended Human-to-Human Transmission during a Monkeypox Outbreak in the Democratic Republic of the Congo. Emerg Infect Dis. 2016 Jun;22(6):1014-21. https://doi.org/10.3201/eid2206.150579 10.3201/eid2206.150579 PMiD: PMCid: 27191380 PMC4880088

[8] Adler H Gould S Hine P. Clinical features and management of human monkeypox: a retrospective observational study in the UK. Lancet Infect Dis. 2022 May 24:S1473-3099(22)00228-6. https://doi.org/10.1016/S1473-3099(22)00228-6 10.1016/S1473-3099(22)00228-6 PMIiD: 35623380 PMC9300470

[9] Vaccines and immunization for monkeypox. World Health Organization. [online] 2022 [cited 2022 June 20]. Available from URL: https://www.who.int/publications/i/item/who-mpx-immunization-2022.1

[10] Belongia EA Naleway AL. Smallpox vaccine: the good, the bad, and the ugly. Clin Med Res. 2003 Apr;1(2):87-92. https://doi.org/10.3121/cmr.1.2.87 10.3121/cmr.1.2.87 PMiD: PMCid: 15931293 PMC1069029

